# Emergence timing and fitness consequences of variation in seed oil composition in *Arabidopsis thaliana*

**DOI:** 10.1002/ece3.1265

**Published:** 2014-12-10

**Authors:** Sandra E Pelc, C Randal Linder

**Affiliations:** 1 US Vegetable Laboratory, Agricultural Research Service, United States Department of AgricultureCharleston, South Carolina, 29414; 2 Department of Integrative Biology, University of Texas at AustinAustin, Texas, 78712

**Keywords:** Adaptive evolution, *Arabidopsis thaliana*, emergence timing, fatty acids, relative fitness, seed oil composition

## Abstract

Early seedling emergence can increase plant fitness under competition. Seed oil composition (the types and relative amounts of fatty acids in the oils) may play an important role in determining emergence timing and early growth rate in oilseeds. Saturated fatty acids provide more energy per carbon atom than unsaturated fatty acids but have substantially higher melting points (when chain length is held constant). This characteristic forms the basis of an adaptive hypothesis that lower melting point seeds (lower proportion of saturated fatty acids) should be favored under colder germination temperatures due to earlier germination and faster growth before photosynthesis, while at warmer germination temperatures, seeds with a higher amount of energy (higher proportion of saturated fatty acids) should be favored. To assess the effects of seed oil melting point on timing of seedling emergence and fitness, high- and low-melting point lines from a recombinant inbred cross of *Arabidopsis thaliana* were competed in a fully factorial experiment at warm and cold temperatures with two different density treatments. Emergence timing between these lines was not significantly different at either temperature, which aligned with warm temperature predictions, but not cold temperature predictions. Under all conditions, plants competing against high-melting point lines had lower fitness relative to those against low-melting point lines, which matched expectations for undifferentiated emergence times.

## Introduction

Timing of germination and seedling emergence can contribute significantly to the overall lifetime fitness of plants, particularly annuals. Many studies have found evidence of a selective advantage to early emergence, especially under competition (Miller et al. 1994; Dyer et al. 2000; Geber and Griffen 2003; Mercer et al. 2011). Seed oil composition (the types and relative amounts of fatty acids in the oils) may play an important role in determining the timing of germination and/or emergence for the 80% of flowering plants that store oils for energy in their seeds (Linder 2000). It has been hypothesized that the melting points of the triacylglycerols in seeds influence the timing of germination and the rate of early growth in plants because lower melting point compositions will be more available for metabolism at cooler temperatures than higher melting point compositions. Consistent patterns in seed oil melting points have been found across the angiosperms along a latitudinal gradient at both the family and the genus levels and within broadly distributed species. Across 680 oilseed species, tropical species had significantly higher proportions of saturated fatty acids in their oils on average than temperate species. This pattern was demonstrated to have evolved independently a minimum of sixteen times (Linder 2000). Oil composition within the wide-spread genus *Helianthus* was significantly negatively correlated with latitude (Linder 2000). The oilseed species, *Plukenetia volubilis* L., had significantly higher proportions of unsaturated fatty acids at higher altitudes (Cai et al. 2012), which would be predicted because temperatures decrease at higher altitudes. These repeated patterns could be explained by selection acting on the relative proportions of fatty acids (Linder 2000).

An adaptive hypothesis to explain this pattern suggests that under competition, selection has acted to modify seed oil composition through a trade-off between total amount of energy stores in seeds and the rate at which that energy can be used at different germination temperatures (Linder 2000). Saturated fatty acids are less costly to produce and provide more energy per carbon atom (Lehninger 1993) but have much higher melting points than unsaturated fatty acids of the same chain length (Eckey 1954; Hilditch and Williams 1964). Seed oils are stored in oil bodies which consist of triacylglycerols surrounded by a single layer of phospholipids with embedded oleosins (Huang 1992). These seed oils provide the energy to fuel emergence and seedling growth prior to the onset of photosynthesis (Harwood 1980; Hayashi et al. 1998; Germain et al. 2001). Higher melting points would cause the oil bodies to be more viscous, slowing Brownian motion and ultimately the chemical reactions necessary for fatty acid *β* oxidation and gluconeogenesis (Kramers 1940). Therefore, oilseeds with higher proportions of unsaturated fatty acids should have lower melting points and should be able to emerge earlier and/or grow more rapidly under colder germination temperatures than competitors with a lower proportion of unsaturated fatty acids in their seed oils. Under warmer germination temperatures, this timing advantage should disappear, and the higher amount of total energy, in the form of a higher proportion of saturated fatty acids, should be favored. Using *Helianthus annuus* seed having high or low amounts of saturated fatty acids in their seed oils, a significant difference in germination timing was not found at a warm germination temperature, while at lower germination temperatures, the seeds with low amounts of saturated fatty acids germinated significantly earlier and grew more rapidly (Linder 2000). This experiment did not test for the expected differences in fitness.

Natural populations of *Arabidopsis thaliana* experience a broad range of temperatures and other climatic conditions across the species' native Eurasian range (Lasky et al. 2012) providing the opportunity for selection on life history characters within different populations. Previous studies have found genetic variation in *A. thaliana* for life history traits, for example, seed dormancy flowering time (Debieu et al. 2013) and have shown that this variation can affect lifetime fitness (Donohue et al. 2005; Huang et al. 2010). The proportions of saturated fatty acids in *A. thaliana* seed oils have been shown to follow a latitudinal cline, with an inverse relationship (Sanyal and Linder 2013). With short generation times and small stature, the weedy annual *A*. *thaliana* is well suited to studies of life history traits in large-scale growth chamber experiments. In this study, we investigated the impact of temperature on time to emergence in four *A. thaliana* recombinant inbred lines (RILs) with disparate seed oil melting points (two with high melting points and two with low) and measured relative fitness. Under the hypothesis of Linder (2000), we had two expectations. First, under lower germination temperatures, lines with lower melting points would have higher relative fitness under competition with a higher melting point line due to earlier emergence than high-melting point lines under competition with lower melting point lines. Second, under warm germination temperatures, we expected no difference in emergence timing between high- and low-melting point lines and for the higher melting point lines to either have similar or higher fitness relative to the lower melting point lines when in competition with individuals of the opposite type. We tested these hypotheses using fully factorial intra (high vs. high or low vs. low) and intertype (high vs. low or low vs. high) competition experiments under controlled temperature conditions using fruit count (number of siliques) as the proxy for fitness.

## Materials and Methods

### Plant materials and seed production

We used four lines from a previously described set of 100 recombinant inbred lines (RIL set CS1899), generated from a cross between *A. thaliana* accessions Columbia (CS933) and Landsberg erecta (CS20; Lister and Dean 1993). Because the seed oil melting points of nearly 100 *A. thaliana* accessions (Sanyal and Linder, 2012) were more strongly correlated with latitude than were saturated fatty acid proportions (data not shown), the RILs for this study were chosen based upon seed oil melting point differences. Melting points were estimated from the RIL set's published seed oil compositions (Sanyal and Randal Linder 2012) by calculating an average of the melting points of the fatty acids, weighted by their relative proportions (Malkin 1954).Two lines were chosen from the upper and two from the lower tails of the distribution of seed oil melting points. The low-melting point lines (L) had melting points of 9.9°C (CS1975) and 10.0°C (CS1984), while the high-melting point lines (H) had melting points of 14.5°C (CS1948) and 14.6°C (CS1994).

Seeds for the RIL set, previously purchased from the Arabidopsis Biological Resource Center (ABRC) and phenotyped for fatty acid proportions by Sanyal and Linder (2012), were grown to produce bulk seed for the four lines chosen for this experiment. To break dormancy, seeds were surface-sterilized and cold stratified in water for 10 days at 4°C prior to planting. Plants were grown under common garden conditions of 15 h light at 22°C and 9 h of dark at 18°C. Moist soil was maintained through subirrigation, and plants were fertilized once a week with Peters professional 20-20-20 (Everris NA, Inc., Geldermalsen, The Netherlands). Upon bolting, plants were kept reproductively isolated using the Arasystem (Betatech, Gent, BE). Seeds were harvested from each plant over a week-long period after the siliques matured and turned brown. The seed produced from these plants was stored at room temperature in unsealed coin envelopes for 20 months prior to the start of this experiment, which exceeded the after-ripening requirements of both parental lines (Alonso-Blanco et al. 2003; Leymarie et al. 2012).

### Experimental design

A fully factorial randomized-block design was implemented for competition trials under two germination temperatures (Cold: 11°C day/6.5°C night and Warm: 16°C day/11°C night) and at two densities (High: 1 cm between the focal and associates, and Low: 3 cm). Cold temperatures were chosen so that low-melting point lines would be liquid/low viscosity during day temperatures, but the high-melting point lines would not. Warm temperatures were higher than the melting point of all four lines to reduce seed oil viscosity differences between the four lines. Following stratification, 3–5 seeds per position were sown into either 2.5-inch-diameter pots (high-density treatment) or 3-inch-diameter pots (low-density treatment). Pots were filled with Metro-Mix 360 (SunGro Horticulture Canada Ltd., Vancouver, BC, Canada). Competition pots consisted of a focal plant surrounded by four competitors of one genotype (high or low melting point). The focal was sown into the middle of the pot, and the competitors were added to the four corners of the pots at distances of 3 cm from the focal in the low-density treatment and 1 cm in the high-density treatment. All possible combinations of focal genotype with competitor genotype were planted (four lines × four lines = 16 combinations). No-competition controls (focal plant only) for each of the four lines were planted in both pot sizes to test whether the density treatments induced competition. Seeds were sown onto the surface of eight replicate pots for each factorial combination of each planting combination for a total of 640 pots (two densities × two temperatures × 20 planting combinations × eight replicates). After establishment, each position was thinned to one seedling. Pot position was randomized across five trays of 2.5″ pots and eight trays of 3″ pots in two growth chambers for a total of 26 trays (13 per growth chamber). Tray was treated as a block, and tray positions were randomized twice per week to minimize position effects.

The temperature treatments were applied during the germination and establishment phase in two different growth chambers. The light intensity was equivalent in both chambers and lights were on 14 h per day.

**Figure 1:**
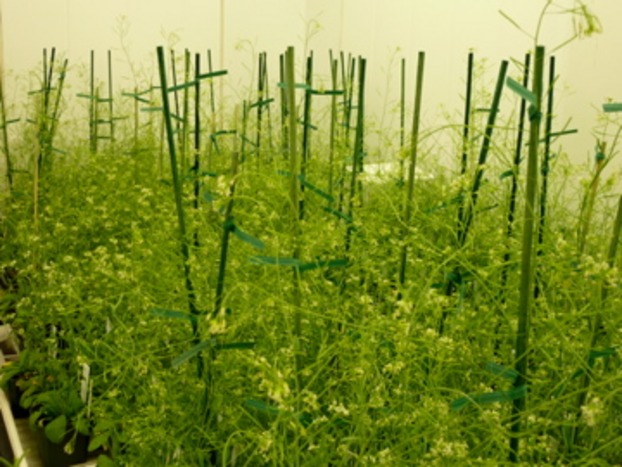
Flowering of *Arabidopsis thaliana* plants in the growth chamber during the competition experiments.Photograph by Sandra E. Pelc.

Measurements were recorded for only focal plants. Pots were observed daily and date of emergence recorded when cotyledons appeared. In the case of *A. thaliana,* emergence timing is a reasonable proxy for germination timing because the seeds are on the soil surface and emergence of the cotyledons quickly follows germination. After no new focals had emerged over a 5-day period, plants were moved to a single growth chamber to finish their life cycle under common garden conditions of 16 h of light at 23°C and 8 h of dark at 18°C (Fig.1). Trays were subirrigated at a frequency that maintained moist soil in pots and were fertilized once per week with Peters professional 20-20-20 (Everris NA, Inc.). A 9-week growing season was enforced by discontinuing watering and removing newly flowering meristems. To assess relative fitness, total fruits (siliques) per focal plant that survived to reproduction were counted. Nineteen focal plants died before reproduction due to fungal infection (no more than 2 replicates per treatment combination) and were excluded from the fitness analyses.

### Statistical analyses

Because the adaptive hypothesis of Linder (2000) makes predictions about emergence timing and relative fitness specific to each temperature treatment, data from the two temperatures were analyzed with separate models. The effects on days to emergence of focal type (H or L), density, and their interaction (all incorporated as fixed effects) were assessed using generalized linear mixed models (proc GLIMMIX in SAS 9.2; SAS Institute, Inc., Cary, NC, USA). The best fit distribution for emergence days in the cold model was Gaussian, and Poisson for the warm model.

To analyze the effects of focal/competitor type, density, and their interaction (all incorporated as fixed effects) on fruit count, proc GLIMMIX was again used but with a negative binomial distribution for both cold and warm models. Orthogonal contrasts were added to the fruit count models to test the hypotheses of Linder (2000). Contrasts of intra and intertype fruit counts for a given focal type (ex. LH vs. LL) were used to address the specific relative fitness predictions of Linder (2000) in both the warm and the cold temperature models. Under cold temperatures, the adaptive hypothesis (Linder 2000) predicts higher relative fitness for high-melting point lines under intratype (HH) competition compared to intertype (HL), and higher relative fitness for low-melting point lines under intertype competition (LH) compared to intratype (LL). Under warm temperatures, focal plants (regardless of type) are predicted to have higher fitness when in competition with low-melting point lines relative to those in competition with high-melting point lines.

## Results

### Time to emergence

**Figure 2:**
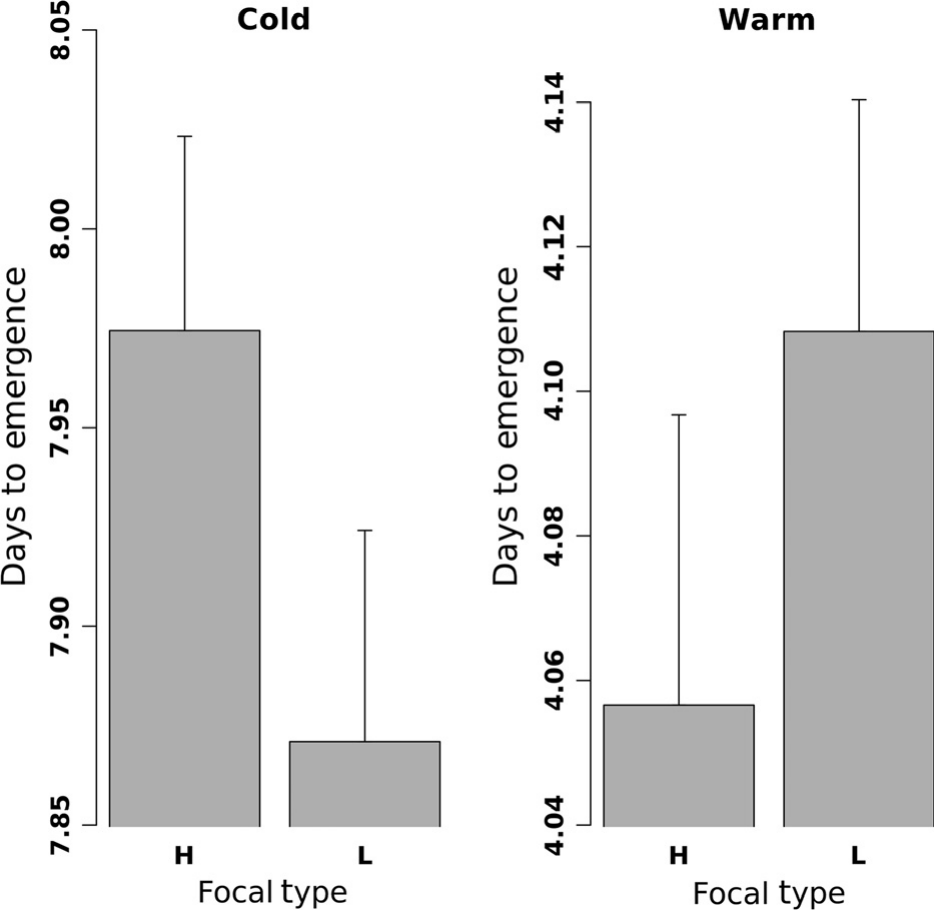
Means and standard errors of days to emergence for the focal plants of the high- (H) and low-(L) melting point lines under cold and warm temperatures.

Temperature had a strong effect on time to emergence, with an average time of 7.9 ± 0.036 days (mean ± SE) in the cold treatment and approximately half the time in the warm treatment (4.1 ± 0.026 days; Fig.2). In the analyses, which factors were significant did not change whether tray was included as a random variable or not from any of the models for emergence timing or fruit count, so the results excluding tray are presented. As expected, density had no detectable effect on emergence time (Table1), but neither did melting point type (H or L). Within a given temperature treatment, no emergence timing differences were significant. However, on average, low-melting point type focal plants tended to emerge earlier than high-melting point lines under cold temperatures (0.10 days).

**Table 1:** Effect of focal melting point type and density on days to emergence under cold and warm germination temperatures

Effect	df	*F* value	*P* value
Temperature = Cold			
Focal type	1, 307	2.04	0.154
Density	1, 307	1.52	0.219
Focal type^*^Density	1, 307	0.28	0.596
Temperature = Warm			
Focal type	1, 312	0.05	0.818
Density	1, 312	0.20	0.655
Focal type^*^Density	1, 312	0.00	0.959

df, degrees of freedom: numerator, denominator.

### Density treatments induce competition

**Figure 3:**
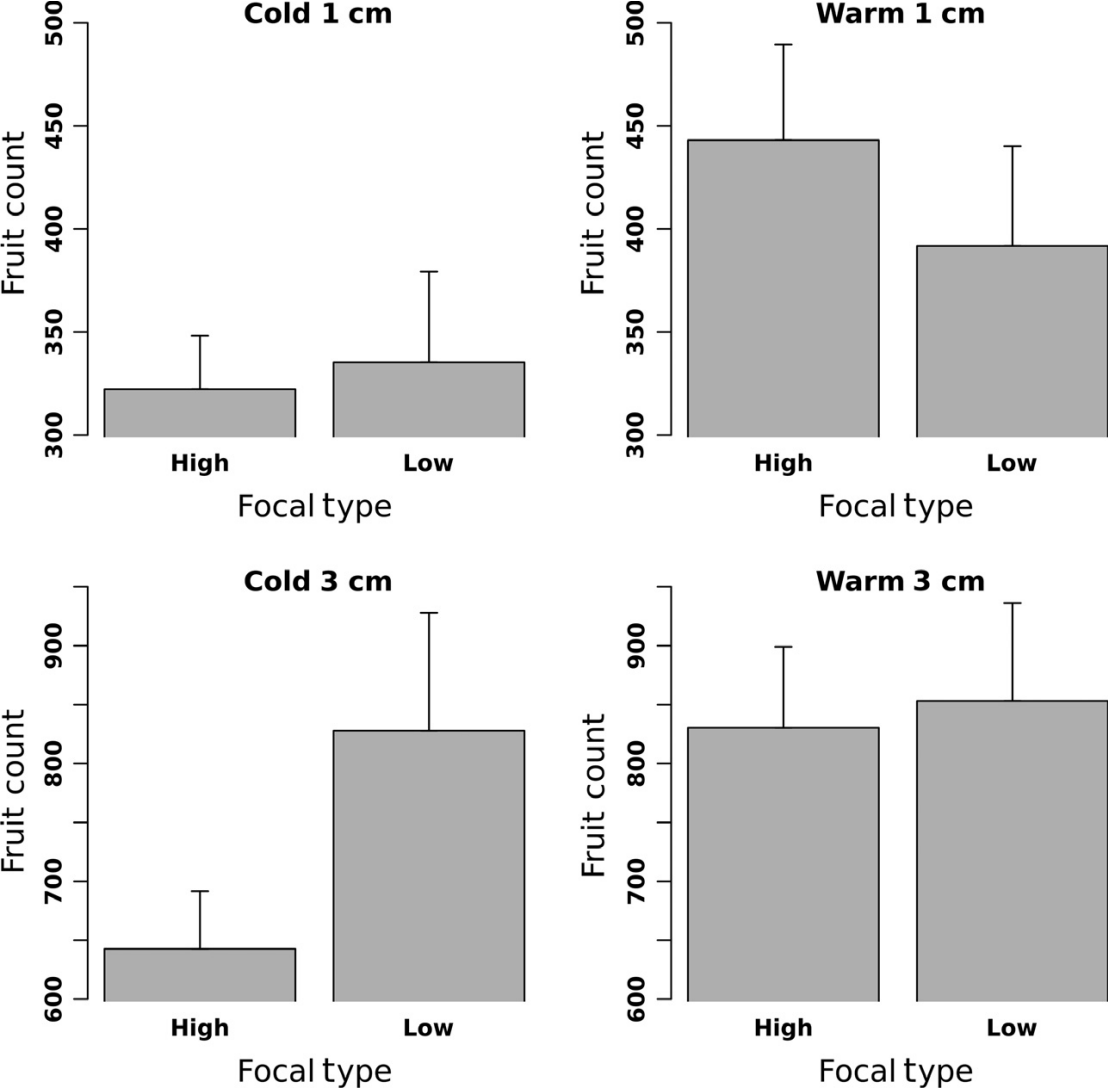
Means and standard errors of fruit count for each focal type (H and L) without competitors. Results are presented for each factorial combination of planting density and temperature. Separate results are presented for each density because no-competition plants were in different size pots depending on the density of the focal grown with competition.

**Figure 4:**
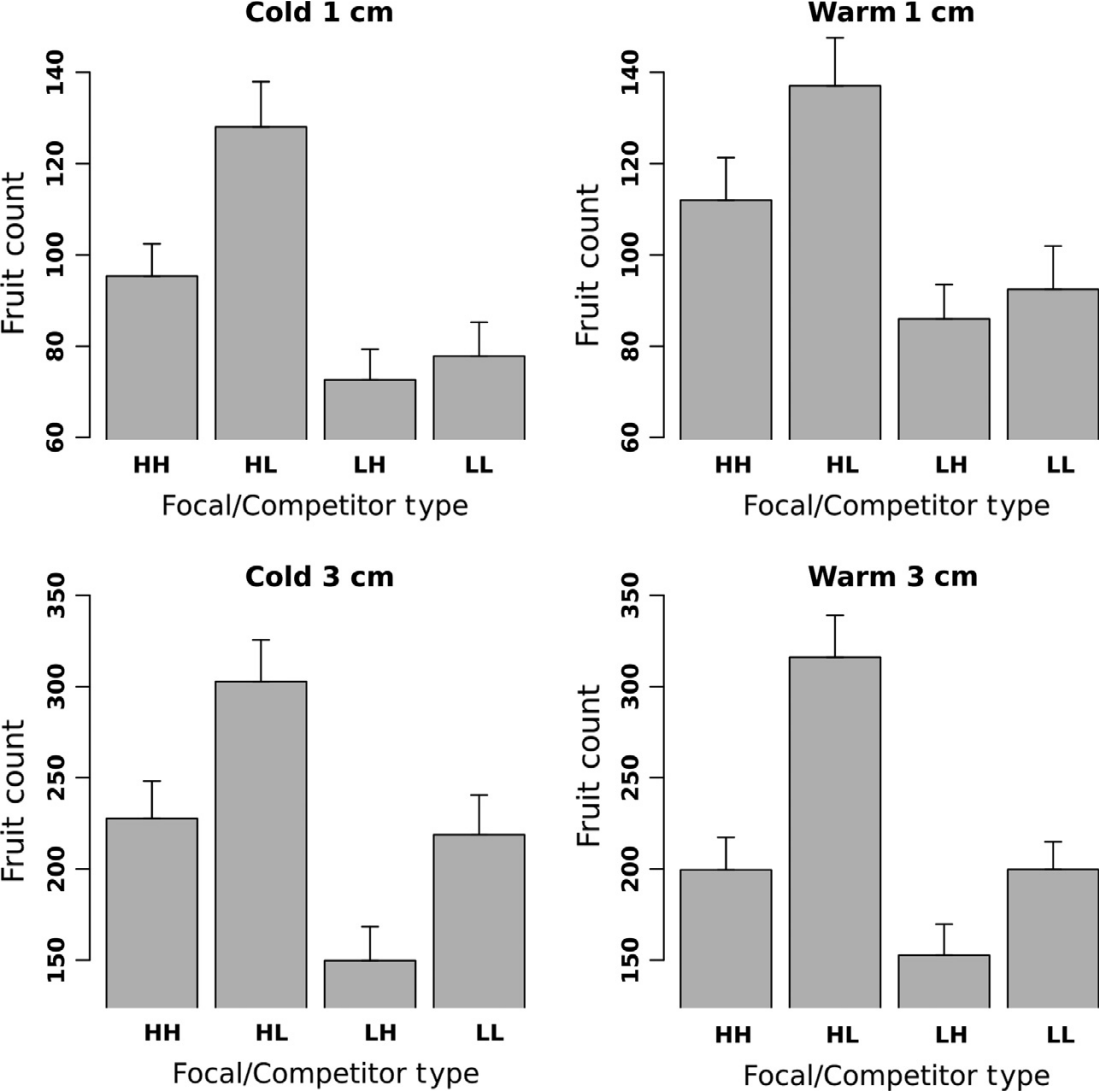
Means and standard errors of fruit count for each Focal/Competitor combination. Results are presented for each factorial combination of planting density and temperature.

For both planting densities (which used different sized pots), the no-competition plants produced significantly more fruits at a given temperature (Fig.3) than the plants with competitors at the same density (Fig.4) demonstrating that we were successful at creating competitive conditions for plants with associates.

High-density no-competition plants grew in smaller pots than the low-density no-competition plants. Pot size had a significant effect on fruit number for no-competition plants, with a mean difference of 414 more fruits per plant for plants growing in the smaller, low-density pots (Fig.3). Overall, within a given density treatment, the no-competition controls produced approximately the same ratio of fruits relative to the plants with competitors (3.7 times more fruits at high density and 3.6 times more at high density). Therefore, even though the high-density competition plants produced an absolute number of fruits that was smaller than the absolute number of fruits in the low-density competition treatment (Fig.4), the relative degree of competition in each density was similar.

### Relative fitness

#### Cold germination temperatures

Under cold germination temperatures, the first expectation that lines with low melting point oils would have a higher relative fitness when in intertype (LH) competition than intratype (LL) competition was not met. There was a significant difference in fruit number produced by the low-melting point lines under the two competition types, but it was in the opposite direction from expectation (*P* = 0.013, Table2). Low-melting point lines had higher relative fitness under intratype competition (150.8 ± 14.9 fruits) than those under intertype competition (111.2 ± 11.0 fruits; Fig.4). There was not a significant interaction between focal/competitor type and density (Table2). The second prediction was that high-melting point lines would have higher fitness under intratype (HH) competition relative to intertype (HL). Here again, the opposite pattern was significant (*P* = 0.001, Table2), with an intertype mean fruit number of 218.4 ± 17.1 and an intratype mean of 162.5 ± 13.8 (Fig.4).

**Table 2:** Effect of Focal/Competitor type and density on fruit count under cold germination temperatures

Effect	df	*F* value	*P* value
Focal/Competitor type (FC)	5, 291	79.39	**<0.0001**
Density	1, 291	216.34	**<0.0001**
Interaction: FC^*^Density	5, 291	0.83	0.5321
Contrast: FC LH vs. LL	1, 291	6.30	0.0126
Contrast: FC HL vs. HH	1, 291	10.94	**0.0011**

H, high-melting point lines; L, low-melting point lines; FC, Focal/Competitor type, where the first letter is the focal type and the second letter is the competitor type. Bold values indicate significant effects.

#### Warm germination temperatures

Under warm germination temperatures, the adaptive hypothesis prediction of higher intratype fitness for low-melting point lines relative to intertype competition was weakly supported by borderline significant contrasts (*P* = 0.052, Table3) with the mean fruit number of intratype competition (145.2 ± 11.3) higher than intertype competition (119.4 ± 10.2).

**Table 3:** Effect of Focal/Competitor type and density on fruit count under warm germination temperatures

Effect	df	*F* value	*P* value
Focal/Competitor type	5, 291	95.36	**<0.0001**
Density	1, 291	148.76	**<0.0001**
Interaction: FC^*^Density	5, 291	0.80	0.5472
Contrast: FC LH vs. LL	1, 291	3.81	0.0519
Contrast: FC HL vs. HH	1, 291	14.88	**0.0001**

H, high-melting point lines; L, low-melting point lines; FC, Focal/Competitor type, where the first letter is the focal type and the second letter is the competitor type. Bold values indicate significant effects.

As expected, at higher germination temperatures, high-melting point lines had significantly higher reproductive success (*P* = 0.0001) when in competition with low-melting point lines (228.0 ± 17.1 fruits) than with the intratype (157.9 ± 11.7 fruits; Fig.4).

## Discussion

Our goal was to determine the effect of seed oil melting point on timing to emergence under different germination temperatures, and the resulting consequences on relative fitness as a test of the adaptive hypothesis that seed oil melting points influence seedling emergence and subsequently plant fitness under competition (Linder 2000).

The only significant difference in timing to emergence was between temperature treatments, with emergence of seeds in the warm treatment in half the time of the cooler temperature. While no difference in emergence timing was expected between seeds with different oil melting points under the warm temperature treatment, we had expected lower melting point seeds to emerge earlier than higher melting point seeds at low temperature. There was a nonsignificant trend of seeds with lower melting points emerging earlier than those with higher melting points, following the direction of predictions. This could be a spurious result or the product of insufficient power to detect a significant difference.

There are at least three possible explanations for the lack of significant emergence timing differences of the high- and low-melting point RIL lines in this study. First, the hypothesis that the latitudinal cline of melting points is driven by selection on germination timing could be incorrect. For example, an alternative hypothesis is that spatially driven population structure has led to suites of traits that vary along latitudinal clines. However, evidence from other work makes this unlikely. It has been found consistently that the relative proportion of saturated fatty acids in seed oils increases at both lower latitudes and altitudes. Across the angiosperms, tropical species on average have a higher proportion of saturated fatty acids than temperate species (Linder 2000), and lower latitude species in the genus *Helianthus* have significantly higher proportions of saturated fatty acids (Linder 2000). This same latitudinal cline of proportions of saturated fatty acids increasing with decreasing latitude has been observed in *A. thaliana* (Sanyal and Linder 2013). Finally, plants from the oilseed species *Plukenetia volubilis* L. collected at higher altitudes had significantly higher proportions of unsaturated fatty acids than plants collected from lower altitudes (i.e., consistently warmer temperatures (Cai et al. 2012)).

In addition to observational evidence from nature, two other experiments have investigated the effect of temperature on germination timing of seeds with different seed oil melting points. In the first, the germination timing of high oleate fad2-2 mutant seeds were compared with wild-type *A. thaliana* seeds below three different germination temperatures (Miquel and Browse 1994). At 6°C and 10°C, the wild-type seeds (lower melting point) germinated significantly earlier than the mutant fad2-2 seeds (higher melting point), while at 22°C germination timing differences were nonsignificant. Additionally, the mutant fad2-2 seeds had a lower rate of germination than the wild-type seeds. It should be noted that the fad2-2 mutant seeds also had reduced total oil content and therefore the effects on germination of content versus composition cannot be fully disentangled. In the second experiment, *H. annuus* seeds with lower proportions of saturated fatty acids germinated significantly earlier than those with higher proportions at cooler temperatures, while the difference of timing was indistinguishable at warmer temperatures (Linder 2000). In this second study, the low-saturated fatty acid and high-saturated fatty acid seeds did not differ significantly by weight. Both of these studies are in accordance with the prediction of Linder (2000) that lower melting point lines will germinate earlier under cold temperatures. In the face of the evidence in support of this adaptive explanation, further testing with appropriate changes in experimental design seems worthwhile.

Second, the nonsignificant trend of earlier emergence of low melting point might be due to insufficient power to reach significance. If that is the case, the relationship is a weak one and one that in this experiment did not produce the expected fitness consequences. Only 2 lines from either end of the melting point distribution of the RIL set were chosen, leading to qualitative melting point factors in the analysis (H vs. L). More power to detect consistent differences in emergence timing would be provided by using more lines so that a quantitative approach to melting point could be incorporated in the model.

Finally, the difference between the melting points of the high and low types may have been too small to have a large effect on germination timing under the temperatures used for this experiment. The cold germination temperature used in this experiment may not have been low enough to produce viscosity changes great enough to cause emergence timing differences between high- and low-melting point lines. The range in melting points of the low-melting point lines was from 9.9°C to 14.6°C, for a 4.7°C difference. We used 11°C daytime/6.5°C nighttime temperatures for the cold treatment, both of the previous experiments used lower cold temperatures, 10°C daytime and 4°C nighttime for Linder (2000) and 10°C and 6°C for Miquel and Browse (1994). Wild accessions of *A. thaliana* have been exposed to natural selection pressures and have a wider range of seed oil melting points (8.7°C–15.8°C) with a difference of 7.1°C and therefore would make a more appropriate study system.

Because significant differences in emergence times were not observed under either temperature treatment, the greater proportions of high-energy saturated fatty acids found in the higher melting point lines might have given them a competitive advantage. Seed oils provide the energy to germinate and grow until at least the onset of photosynthesis and therefore, maximizing this energy source should be beneficial. Because saturated fatty acids provide more energy per carbon atom than unsaturated and are cheaper for the mother plant to produce (Lehninger 1993), it follows that with a given seed size and oil content, more energy can be provided to the growing seedling by filling it with saturated fatty acids. In support of this idea, within every treatment, plants of both types competing against high-melting point lines (14.2% and 14.6% saturated fatty acids) had significantly lower relative fitness than those competing against lower melting point lines (11.5% and 12.1% saturated fatty acids). If this experiment were redone with colder germination temperatures and low melting-point lines emerged significantly earlier than high-melting point lines the fitness differences predicted by Linder (2000) would be expected. The predictions would be that low-melting point lines would have higher intertype (LH) fitness relative to intratype competition (LL) and high-melting point lines would have higher intratype (HH) fitness relative to intertype competition (HL).

## References

[b1] Alonso-Blanco C, Bentsink L, Hanhart CJ, Blankestijn-de Vries H, Koornneef M (2003). Analysis of natural allelic variation at seed dormancy loci of *Arabidopsis thaliana*. Genetics.

[b2] Cai ZQ, Jiao DY, Tang SX, Dao XS, Lei YB, Cai CT (2012). Leaf photosynthesis, growth, and seed chemicals of Sacha Inchi plants cultivated along an altitude gradient. Crop Sci.

[b3] Debieu M, Tang C, Stich B, Sikosek T, Effgen S, Josephs E (2013). Co-variation between seed dormancy, growth rate and flowering time changes with latitude in *Arabidopsis thaliana*. PLoS ONE.

[b4] Donohue K, Dorn L, Griffith C, Kim E, Aguilera A, Polisetty CR (2005). Niche construction through germination cueing: life-history responses to timing of germination in *Arabidopsis thaliana*. Evolution.

[b5] Dyer AR, Fenech A, Rice KJ (2000). Accelerated seedling emergence in interspecific competitive neighbourhoods. Ecol. Lett.

[b6] Eckey EW (1954). Vegetable fats and oils.

[b7] Geber M, Griffen L (2003). Inheritance and natural selection on functional traits. Int. J. Plant Sci.

[b8] Germain V, Rylott EL, Larson TR, Sherson SM, Bechtold N, Carde J (2001). Requirement for 3-ketoacyl-CoA thiolase-2 in peroxisome development, fatty acid beta-oxidation and breakdown of triacylglycerol in lipid bodies of Arabidopsis seedlings. Plant J.

[b9] Harwood JL, Stumpf PK (1980). Plant acyl lipids: structure, distribution, and analysis. The biochemistry of plants: a comprehensive treatise.

[b10] Hayashi M, Toriyama K, Kondo M, Nishimura M (1998). 2,4-Dichlorophenoxybutyric acid-resistant mutants of Arabidopsis have defects in glyoxysomal fatty acid beta-oxidation. Plant Cell.

[b25] Hilditch TP, Williams PN (1964). The chemical constitution of natural fats.

[b11] Huang A (1992). Oil bodies and oleosins in seeds. Annu. Rev. Plant Biol.

[b12] Huang X, Schmitt J, Dorn L, Griffith C, Effgen S, Takao S (2010). The earliest stages of adaptation in an experimental plant population: strong selection on QTLS for seed dormancy. Mol. Ecol.

[b13] Kramers HA (1940). Brownian motion in a field of force and the diffusion model of chemical reactions. Physica.

[b14] Lasky JR, Marais DL, Des McKay JK, Richards JH, Juenger TE, Keitt TH (2012). Characterizing genomic variation of *Arabidopsis thaliana*: the roles of geography and climate. Mol. Ecol.

[b15] Lehninger AI (1993). Biochemistry.

[b16] Leymarie J, Vitkauskaité G, Hoang HH, Gendreau E, Chazoule V, Meimoun P (2012). Role of reactive oxygen species in the regulation of Arabidopsis seed dormancy. Plant Cell Physiol.

[b17] Linder C (2000). Adaptive evolution of seed oils in plants: accounting for the biogeographic distribution of saturated and unsaturated fatty acids in seed oils. Am. Nat.

[b18] Lister C, Dean C (1993). Recombinant inbred lines for mapping RFLP and phenotypic markers in *Arabidopsis thaliana*. Plant J.

[b19] Malkin T, Holman TMRT, Lundberg WO (1954). The polymorphism of glycerides. Progress in the chemistry of fats and other lipids.

[b20] Mercer KL, Alexander HM, Snow AA (2011). Selection on seedling emergence timing and size in an annual plant, *Helianthus annuus* (common sunflower, Asteraceae). Am. J. Bot.

[b21] Miller T, Winn A, Schemske D (1994). The effects of density and spatial distribution on selection for emergence time in *Prunella vulgaris* (Lamiaceae). Am. J. Bot.

[b22] Miquel MF, Browse JA (1994). High-oleate oilseeds fail to develop at low temperature. Plant Physiol.

[b24] Sanyal A, Randal Linder C (2012). Quantitative trait loci involved in regulating seed oil composition in *Arabidopsis thaliana* and their evolutionary implications. Theor. Appl. Genet.

[b23] Sanyal A, Linder C (2013). Plasticity and constraints on fatty acid composition in the phospholipids and triacylglycerols of Arabidopsis accessions grown at different temperatures. BMC Plant Biol.

